# Developing the universal unified prevention program for diverse disorders for school-aged children

**DOI:** 10.1186/s13034-019-0303-2

**Published:** 2019-11-13

**Authors:** Shin-ichi Ishikawa, Kohei Kishida, Takuya Oka, Aya Saito, Sakie Shimotsu, Norio Watanabe, Hiroki Sasamori, Yoko Kamio

**Affiliations:** 1 Faculty of Psychology, Doshisha University, 1-3 Tatara Miyakodani, Kyotanabe, Kyoto 610-0394 Japan; 2 Graduate School of Psychology, Doshisha University, 1-3 Tatara Miyakodani, Kyotanabe, Kyoto 610-0394 Japan; 3 The Japan Society for the Promotion of Science, 5-3-1 Kojimachi, Chiyoda-ku, Tokyo 102-0083 Japan; 4 Department of Preventive Intervention for Psychiatric Disorders, National Institute of Mental Health, National Center of Neurology and Psychiatry, 4-1-1, Ogawa Higasahi-cho, Kodaira, Tokyo 187-8553 Japan; 5 Faculty of Human Development and Education, Kyoto Women’s University, 35 Kitahiyoshi-cho, Imakumano, Higashiyama-ku, Kyoto, 605-8501 Japan; 6 Department of Health Promotion and Human Behavior, Department of Clinical Epidemiology, Kyoto University Graduate School of Medicine/School of Public Health, Yoshida Konoe-cho, Sakyo-ku, Kyoto, Kyoto 606-8501 Japan; 7 Center for Promoting Education for Persons with Developmental Disabilities, National Institute of Special Needs Education, 5-1-1 Nobi, Yokosuka, Kanagawa Prefecture 239-8585 Japan; 8 Center for Institutional Research, Educational Development, and Learning Support, Ochanomizu University, 2-1-1 Ohtsuka, Bunkyo-ku, Tokyo, 112-8610 Japan; 9 Institute for Educational and Human Development, Ochanomizu University, 2-1-1 Ohtsuka, Bunkyo-ku, Tokyo, 112-8610 Japan

**Keywords:** Cognitive-behavioral therapy, Universal prevention, Transdiagnostic, School, Children

## Abstract

**Background:**

Psychological problems during childhood and adolescence are highly prevalent, frequently comorbid, and incur severe social burden. A school-based universal prevention approach is one avenue to address these issues.

**Objective:**

The first aim of this study was the development of a novel, transdiagnostic cognitive-behavioral universal prevention program: The Universal Unified Prevention Program for Diverse Disorders (Up2-D2). The second aim of this study was to examine the acceptability and fidelity of the Up2-D2.

**Methods:**

Classroom teachers who attended a 1-day workshop implemented the Up2-D2 independently as a part of their regular curricula. To assess the acceptability of the Up2-D2, 213 children (111 boys and 102 girls) aged 9–12 years completed questionnaires about their enjoyment, comprehension, attainment, applicability, and self-efficacy after completing Lessons 1–12. For fidelity, research assistants independently evaluated audio files that were randomly selected and assigned (27.3%).

**Results:**

Our preliminary evaluation revealed the program was highly enjoyable, clear, and applicable for students. In addition, self-efficacy demonstrated a trend of gradually increasing over the 12 sessions. The total fidelity observed in the two schools was sufficient (76.2%), given the length of the teacher training.

**Conclusions:**

The results of this study supported the theory that the Up2-D2 could be feasible in real-world school settings when classroom teachers implement the program. We discussed current research and practical issues of using universal prevention to address mental health problems in school, based on implementation science for user-centered design.

## Background

Contrary to widespread belief, mental disorders are common during childhood and adolescence with 10–20% of all children experiencing one or more of these problems, incurring severe social burden; consequently, mental health promotion is an urgent issue, and early detection and intervention are essential [1]. Moreover, a recent meta-analysis estimated that the worldwide prevalence of mental disorders was 13.4% (95% confidence interval 11.3–15.9) among a sample of 87,742 children [2]. This suggests that approximately 241 million youths are affected by at least one mental disorder globally.

Although fear and anxiety are considered normal emotions that every child experiences during typical development, some children have profoundly high anxiety levels compared to typically developing children, which can cause severe impairment in their daily lives. Anxiety disorders are the most common psychological problem among children and adolescents [2, 3]. Moreover, anxiety disorders in children and adolescents predict mental health difficulties broadly in their later life including anxiety disorders, mood disorders, and substance abuse [4].

Children and adolescents are also currently experiencing depression at an unprecedented rate [5]. Recently, prevalence studies in Japan have shown that 8.8% of adolescents aged 12–14 years met one or more depressive disorders based on the *Diagnostic and Statistical Manual of Mental Disorder*s, *Fourth Edition, Text Revision* [6]. Depression in children and adolescents often co-occurs with anxiety disorders [7]. Furthermore, anxiety and depression are also frequently occurring in children and adolescents with neurodevelopmental disorders such as attention-deficit/hyperactivity disorders (ADHD) or autism spectrum disorder.

Finally, anger and irritability are relatively common behaviors in children and adolescents aged 9 to 16 years (51.4% showed phasic irritability in a community sample) [8] and are the most frequent reasons for mental health referrals [9]. Although anger/irritability is a core symptom of oppositional defiant disorder or disruptive mood dysregulation disorder, irritability is also seen in children with anxiety disorders, depressive disorders, or ADHD [10]. Children and adolescents frequently experience a wide variety of emotional and behavioral difficulties throughout their development. Regardless of whether the severity of these issues meet the clinical criteria for a diagnosis, preventive interventions can support behavioral and emotional regulation related to a wide variety of concerns, ultimately promoting positive youth development and even mitigating the onset or severity of later disorders.

### Preventative actions in schools

Since students learn and develop their social and emotional competence in school, schools play a key role in fostering healthy social and emotional development among youths [1]. Specifically, teachers, as models, are in a very powerful position and their opinions concerning what constitutes mental health impacts the concepts of mental health adopted by their students [11]. School-based approaches, especially those implemented by schoolteachers, are a crucial avenue for the prevention of mental health problems [12].

Diverse school-based prevention programs have been developed and examined in several countries. There are three types of school prevention programs: universal, selective, and indicated [13]. Universal prevention includes all members regardless of their risk status. Selective prevention focuses on individuals who have a risk for mental disorders, such as parental psychopathology or adverse circumstances. Indicated prevention means an intervention for individuals who already have mild to moderate symptoms.

Among the three types of prevention programs, universal prevention in school has several inherent advantages. First, a universal prevention program can access most students who are enrolled in each school district, while rarely experiencing attrition. Second, a universal approach can minimize the risk of “labeling” for students who are removed from a classroom for selective or indicated interventions. Third, a universal approach can strengthen the protective role of the school environment, which might have proximal influences on children, according to the ecological model of child mental health [14]. Fourth, because all students can participate regardless of risk or diagnostic status, implementation of a universal prevention program can support future selective and/or indicated interventions as a framework for layered or stepped preventive approaches. Universal prevention based on a cognitive-behavioral approach is designed to enhance individuals’ specific coping strategies for current/future adversity, and encourages application of those skills to support other students. A previous trial for adult outpatients with anxiety and depressive symptoms suggested that group cognitive-behavioral therapy (CBT) can ameliorate their emotional symptoms as well as improve their self-stigma [15]. A group-based CBT in the classroom showed increased knowledge about mental health and decreased stigma to individuals with mental disorders. Moreover, students in the 5th and 6th grades who participated in the intervention showed significant improvement in self-efficacy, indicating that they can support friends and people around them with mental health problems [16]. Therefore, students, as well as school personnel, can acquire mental health literacy and reduce stigma for mental disorders through teaching cognitive-behavioral skills.

### Evidence of prevention programs in schools

Most school prevention programs for mental health were based on cognitive-behavioral interventions [17]. Some were created as universal programs, whereas others were originally designed for selective or indicated programs. For example, open trials for universal depression prevention interventions have shown a significant improvement in social skills and a reduction in depressive symptoms among elementary school children aged 8 to 12 years [16, 18], and the positive effect was maintained three years later [19].

Several systematic reviews of school-based prevention programs for depression covering ages ranging from 5 to 22 years old have been published [20–22]. These studies showed that targeted (i.e., selective and indicated) programs could be marginally superior to universal prevention programs, while the efficiency of universal prevention programs was somewhat inconsistent. The Cochrane Review in 2011 affirmed some evidence that universal, as well as targeted depression, prevention programs may prevent the onset of depressive disorders compared with no intervention in children and adolescents aged 5 to 19 years [23]. However, the latest review of depression prevention programs concluded that prevention programs delivered universally to child and adolescent populations aged 5 to 19 years showed “a sobering lack of effect when compared with an attention placebo control” ([24] p. 49).

Regarding anxiety, Neil and Christensen [25] reviewed 27 randomized controlled trials of school-based programs for children (5–12 years) or adolescents (13–19 years). Over half the studies (59%) were universal prevention programs (30% were indicated programs and 11% were selective programs). Approximately eleven of the sixteen (69%) universal trials reported significant improvement post-intervention (ES = 0.31 to 1.37), while five trials failed to find significant improvement (ES = − 0.21 to 0.28). According to a meta-analysis of school-based prevention programs focused on both anxiety and depression for kindergarten through 12th grade, including 31 universal trials [26], there was no clear effect for anxiety; however, a significant improvement for depression was shown in a direct comparison between intervention and control participants (*Z*s = 0.99 and 2.77, respectively, *p* < 0.01). Whereas universal preventive actions for anger and anger-related problems have been addressed as being useful to improve children’s social and academic development in kindergarten and early childhood [27], there is no research using CBT-based universal prevention programs for anger-related problems in middle to late childhood (6–18 years) [28]. Therefore, despite its promising results and partial support for its effectiveness, there is room for improvement in universal prevention research, especially concerning the magnitude of its effects.

### The current research tasks for universal prevention programs in schools

Previous studies suggested two issues that should be addressed in future studies of universal prevention programs in school: (1) to optimize inherent advantages of universal prevention in school overcoming limited effects, and (2) to explore the user-centered design of a universal prevention program for enhancing participants’ motivation that might facilitate more reliable gains.

Recently, a transdiagnostic approach is gathering much attention. This approach can address comorbidities frequently seen in clinical populations and redundancies of learning distinct treatment manuals for practitioners [29, 30]. There are three types of transdiagnostic approaches: the core dysfunction approach, common elements approach, and principle-guided approach [30]. First, the core dysfunctional approach addresses multiple psychological problems by targeting underlying common dysfunction. As a typical example, the *Unified Protocol for the Transdiagnostic Treatment of Emotional Disorders* (UP) [31] shows the frequently used approaches include treatment for problems that possess overlapping etiology, underlying shared pathological processes, or maintaining common processes [32]. Therefore, whereas it might be one of the first-line options for anxiety and depression, it needs further consideration to expand its utilization to more diverse disorders. Second, a common elements approach intends to select as many as common components that are derived from empirically supported treatments designed for distinct disorders. The approach may be workable when the elements can be compiled as separable, independent, and structured components [30]. Given that classroom teachers are used to teaching structured components in the classroom, the approach might be advantageous for universal prevention in schools. On the other hand, a flexible approach which allows therapists to use these components discreetly is not adequate for universal prevention programs. Third, the principle-guided approach possesses a high level of flexibility for intervention content and sequencing based on therapists’ clinical decisions. Therefore, the principle-guided approach might be efficacious for clinical settings due to its flexibility; however, it is also difficult to apply to the universal prevention protocols that are implemented by schoolteachers.

As mentioned, previous studies regarding school-based preventive CBT programs have focused on a single type of psychopathology. However, CBT programs among clinical populations can produce diverse therapeutic gains for a variety of psychological disorders that are often co-occurring in a child or adolescent [33]. Given that CBT was originally conceived as a broad paradigm for treating psychological disorders [34] and that the current components of empirically supported treatments for internalizing and externalizing disorders are largely shared [35], a universal prevention approach based on CBT might be effective for diverse mental health domains using a transdiagnostic approach. To the best of our knowledge, no research has examined CBT’s applicability in universal preventive approaches, although several trials of targeted programs are in progress [36, 37]. Even if a transdiagnostic approach is promising, it is essential to determine which design would be suitable for, and applicable to, universal prevention programs in schools. A universal prevention program might inherently reduce motivation for attendance due to the diffusion of its focus. Therefore, we should consider these aspects during the development phase, a priori, since research is often concerned with adaptation and implementation after completion of efficacy studies [38]. Specifically, (a) as previously stated, some efficacy trials of universal prevention programs targeting a single psychological problem failed to show clear evidence according to the rigorous criteria; (b) however, each program targeting a single psychological problem included evidence-based components derived from CBT, which is strongly empirically supported; and (c) we should explore if an entirely new transdiagnostic universal program that can be applied to diverse children and adolescents in actual school settings.

### Study purpose

To tackle these issues, first, we developed a new school-based universal prevention program—the *Universal Unified Prevention Program for Diverse Disorders* (Up2-D2), which targets transdiagnostic mental health problems based on a cognitive-behavioral approach in schools. Our second purpose was to examine the acceptability and fidelity of the Up2-D2 in school settings after schoolteachers implemented the Up2-D2. Since the acceptability and fidelity of the program should be confirmed in real school settings, classroom teachers and their students evaluated the implementation of the Up2-D2 rather than researchers and clinicians.

## Development of the Up2-D2

The Up2-D2 aims at broad-band effects on mental health problems in elementary and junior high school (i.e., middle school) students aged 8–15 years. The Up2-D2 was designed to integrate common components in CBT for children and adolescents based on evidence-based psychosocial interventions [35] such as psychoeducation, behavioral activation, social skills training, relaxation, cognitive restructuring, gradual exposure, and problem-solving (Table 1). As mentioned in detail below, we modified and adjusted these components to a school curriculum as well as an educational format so that classroom teachers can run the program in their classroom, which was based on previous evidence [39].

**Table 1:** Components of the Up2-D2

No.	Aim	Component	Summary
1	Introduction of the program	Psychoeducation	Starting the program, confirmation of the rules, introduction of characters, explanation about inventions (cognitive-behavioral skills), and program orientation
2	Exploring pleasant events	Behavioral activation	Finding pleasant activities that students can enjoy and exploring other activities that student can engage in even when feeling depressed
3	Learning about kind words	Social skills training	Learning and training how to communicate with peers by using kind words through verbal instruction, modeling, behavioral rehearsal, feedback, and homework
4	Learning about assertive skills	Social skills training	Learning and training how to communicate with peers by use of assertive skills through verbal instruction, modeling, behavioral rehearsal, feedback, and homework
5	Relaxation training	Relaxation	Identifying physical symptoms as a sign for psychological distress; understanding connection between psychological and physical symptoms; and exploring and training their own relaxation, such as progressive muscle relaxation and abdominal breathing techniques
6	Identifying one’s own and others’ strengths	Strength work	Exploring strengths of everyone, understanding differences in individuals, and identifying one’s own and others’ strengths
7	Discovery of own cognition	Cognitive restructuring	Examining the relationship between situation and emotions, finding cognitions between them, and discovering one’s own thoughts
8	Challenging unhelpful thoughts	Cognitive restructuring	Understanding unhelpful thoughts that lead to emotional problems, identifying one’s own typical unhelpful thoughts, and challenging these unhelpful thoughts
9	Preparing behavioral challenges	Exposure	Understanding differences in individuals’ difficulties, identifying the theme of challenging, and understanding the exposure mechanisms
10	Building-up behavioral challenges	Exposure	Making up one’s own hierarchy, discussing how to attempt small challenges, and planning behavioral challenges
11	Learning about problem-solving skills	Problem solving	Introducing steps for problem solving, thinking about solutions as much as possible, evaluating each solution based on multiple criteria, and trying to select the best solution
12	Conclusion	Review and conclusion	Reviewing learned skills (inventions), discussing how to combine these skills and apply daily adversities, and graduation ceremony

*Up2*-*D2* the Universal Unified Prevention Program for Diverse Disorders

**Fig. 1:**
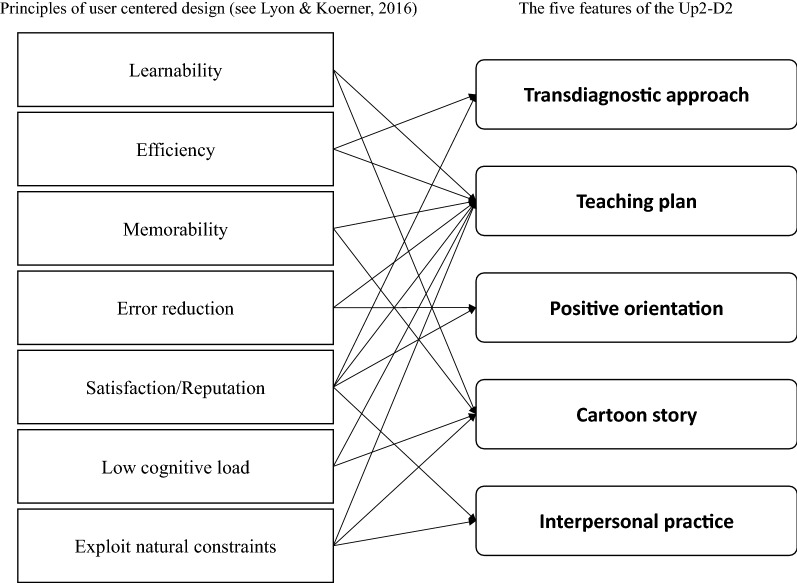
The relationships between principles of user-centered design of evidence-based practice and features of the Up2-D2

One of the fields of research should be the implementation and promotion of the systematic adoption of research findings and other evidence-based practices into routine practice; thus, research focusing on implementation could improve the quality and effectiveness of mental health services [40]. To achieve the application of the research findings, the Up2-D2 was created to examine the principle of a user-centered design for the evidence-based practice. In line with these concepts, Lyon and Koerner [41] conceptualized seven elements for ensuring its usability and effective implementation of the packages that were originally developed by researchers outside of the field. These programmatic concepts were applied for the purpose of this study. The first concept, *learnability*, means that the developer should consider how to build understanding rapidly and easily for teachers and students from the program. Second, *efficiency* refers to the idea that a school-based program should minimize the time, effort, and cost of its usage for addressing targeted problems. Third, *memorability* suggests that a program should be designed to maximize competencies in teachers and students for remembering core elements of CBT. Fourth, *error reduction* aims to prevent error and ensure rapid recovery from the misuse and misunderstanding by use of refinement and elaboration of design. Fifth, *satisfaction*/*reputation* refers to developing a program that should be acceptable, valuable, and attractive for all related users including principals, administrators, teachers, parents, and students in the school. Sixth, a *low cognitive load* means that the developer should focus on simple activities, as well as developing a structure that would be welcomed by the school in order to minimize the cognitive load. Seventh, a program that intends to *exploit natural constraints* is one that should be designed to fit their context of use and maximize existing circumstances and natural contexts. Figure 1 illustrates the correspondence between the seven principles of the user-centered design and the five features of the Up2-D2: transdiagnostic approach, teaching plan, positive orientation, cartoon story, and interpersonal practice.

### Transdiagnostic approach

A transdiagnostic approach is one of the avenues to achieve the goals of efficiency and satisfaction/reputation in addition to enhancement on coverage of diverse mental health problems. Teachers can efficiently administer the unified program targeting multiple problems instead of spending more time conducting multiple programs targeting a single problem. Reducing training load, especially for novices, is one of the inherent benefits related to efficiency in the transdiagnostic approach [42]. Moreover, a program that can cover both internalizing and externalizing problems of students might be highly acceptable for school personnel considering Japanese educational needs. A previous study that examined depression prevention programs in schools noted that feedback from teachers stated that they need more comprehensive programs that can deal with externalizing and internalizing problems [39]. Further, a recent national survey in Japan revealed the worst rates of school refusal, violence, and bullying in elementary schools in recorded history and suggested complex mental health problems may underlie such school issues [43].

### Teaching plan

We created a “teaching plan” for all lessons concerning all elements for effective implementation in schools: learnability, efficiency, memorability, error reduction, low cognitive load, satisfaction/reputation, and exploit natural constraints. In Japan, all academic classes such as mathematics, English, and science are taught based on teaching plans. In addition, they can be optimized depending on each class in accordance with the guidelines provided by the Ministry of Education. Therefore, teaching plans are subject to limitations in existing resources and time-limited opportunities in educational settings. In the teaching plans, every psychological term used in the treatment manual was carefully translated into commonly used expressions in the educational settings to enable teachers to learn the components of the Up2-D2 efficiently and effortlessly. A teaching plan describes all procedures of each lesson of the Up2-D2, which guides teachers to engage in a school-based CBT (Table 2). The plan shares common steps through Lessons 1 to 12 including an introduction, target skills, practice, and conclusion. Since the steps were consistent with the regular curricula, it was also profitable for teachers to comprehend the outline of each lesson, capture the objectives of specific techniques, and monitor the progress of both what they and their students understood. Therefore, preparation of the teaching plans is helpful and indispensable for optimizing the integrity of the present program.

**Table 2:** Flow of each lesson for the Up2-D2

Phase	Contents	Description
Introduction	Goal of today’s lesson Confirmation of the rule Review of the last class (after lesson 2)	At the beginning of every lesson, the teacher should mention the program rules, such as do not make fun of someone, do not mess around, and do not be shy. A teacher starts each lesson with an explanation of today’s goal. After Lesson 2, a teacher also reviews and confirms the homework from the last lesson
Target skills	A vignette Introduction of the target skills	A situation with some difficulties or distress is provided to students in the form of a cartoon. There are three children who have distinct problems in the cartoon. Their problems represent anxiety, depression, and anger, respectively. An inventor plays a role of facilitator and he shows his invention, which acts as a metaphor for the target skills
Practice	Individual practice Group activity	First, students practice the target skill individually. Generally, students are told to complete their worksheets. Then, after sharing, students participate in group activities including, discussion, modeling, and/or behavioral rehearsal
Conclusion	Homework Summary and review of the today	A teacher makes conclusive remarks and explains homework for daily practice. Students complete a comprehension and feedback sheet

*Up2*-*D2* the Universal Unified Prevention Program for Diverse Disorders

### Positive orientation

Positive orientation means that teachers and students can participate in the program with a positive mind and a warm atmosphere by using specific materials, activities, and classroom management. The perception that “childhood is cheeriness and naivety,” or the Japanese proverb to “let sleeping dogs lie,” might represent one of the cultural aspects regarding an unwillingness to tackle mental health problems in children, explicitly [39]. A previous survey in Japan suggested that teachers exhibited less knowledge regarding mental health literacy concerning childhood psychological disorders than mental health professionals and graduate students [44]. With this in mind, we shifted the Up2-D2 from pure cognitive-behavioral techniques to focus more on educational interventions that are positively oriented to minimize misunderstandings, enhance the self-efficacy of teaching, obtain more acceptability, and improve the general reputation of school personnel. In addition, we added works where students are encouraged to find both their own and peers’ strengths in the Up2-D2. Such activities that are derived from the positive psychological intervention for classroom [45] will be welcomed to the Japanese educational settings as positive-oriented classwork. Since Japanese individuals tend to emphasize interdependent aspects where a member is expected to consider and sense what others are feeling and thinking [46], students may find it difficult to ponder their differences rather than their commonalities. Even if they do find their differences, they are liable to be reluctant to disclose such discrepancies in front of their class, especially regarding negative thoughts and/or adversities. Rather, through strength work, students will be likely to identify some differences among individuals more smoothly in positive orientation; then, they will work on their difficulties and adversities more naturally. In addition, given the current model of mental health, which encourages assessment of both wellness and illness [47], universal prevention in school should focus on positive mental health promotion as well as the risk factors of psychological disorders. Since previous strength-based school interventions produced positive gains in life satisfaction and positive affects [48], a cognitive-behavioral intervention combined with strength work could promote positive mental health as well as decrease psychopathological problems.

### Cartoon story

**Fig. 2:**
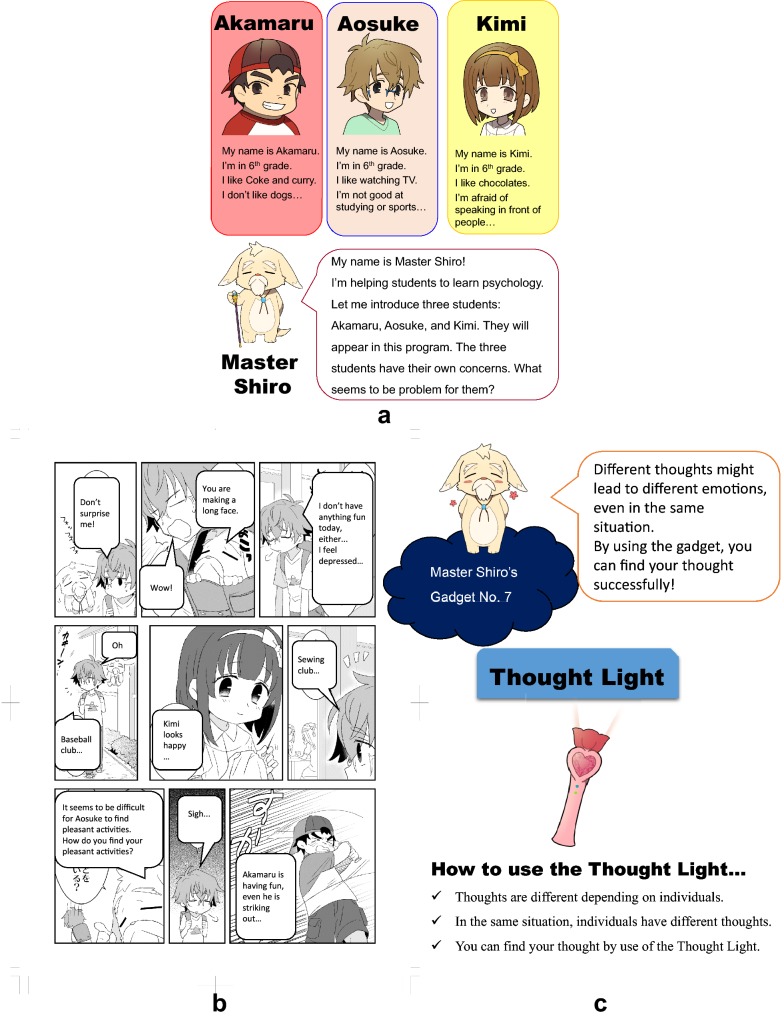
Example of the Up2-D2 illustrations; **a** Three characters and a facilitator; **b** An example of a cartoon story; **c** an example of a gadget

To strengthen the learnability, memorability, low cognitive load, and exploit natural constraints, we created four original cartoon characters: one plays a teacher-like role (a facilitator), and three characters (depressed, anxious, or irritable child) learn skills through lessons (Fig. 2a). The Up2-D2 was developed to have a storyline in which the characters experience distress in a common situation at school and learn how to cope with them (Fig. 2b). First, a common situation with some difficulties or distress in school is shown to students in the target skills section. Through simulating experiences of those characters, students can imagine feeing distressed that they have not experienced so far and can understand how to help their peers overcome such adversities. Second, a target skill that students are expected to learn from the lesson is visualized as a metaphor. This was named a “gadget,” where a facilitator (who is a hermit dog-like animal called “Master Shiro”) provides to the three child characters to help them. It could be useful for students to remember essential points of learned skills with a less cognitive load stimulating intuitive comprehension. For example, “Thought Light” in Lesson 7 was used to represent a skill to identify an individual’s thought (Fig. 2c). Since Japanese students are very familiar with cartoons, learning by use of cartoons can maintain long-term memories and enhance motivation in students [49].

### Interpersonal practice

In addition to the cartoon story, interpersonal practice, another facet of cultural adaptation in the Up2-D2, can ensure the satisfaction and reputation of the program as well as exploit natural constraints (see Table 2). According to a systematic review of recent CBT studies for children and adolescents in Japan [50], group-based interventions especially focused on interpersonal relationships were highly prevalent and were well accepted. In addition, teachers in Japan are clearly encouraged to use group activities as much as they can (especially in “integrated study” and “special activities”). Spence [51] noted that environmental—(e.g., peer support and positive classroom environments), as well as individual-protective factors (e.g., building children’s cognitive-behavioral skills), are essential for universal preventive intervention (i.e., the dual approach) [19]. Although group works and activities are frequently used by the previous programs globally, environmental components should be more underscored and imperative for successful cultural adaptation of CBT originated from Western culture [39]. In the Up2-D2, activities which are usually handled individually like cognitive restructuring (e.g., to find negative maladaptive thoughts or to find more appropriate thoughts) are also reorganized as group ones, given the interdependency of Asian culture [46].

### A preliminary implementation: acceptability and fidelity of the Up2-D2 in schools

We examined the acceptability and fidelity the Up2-D2 when schoolteachers implemented this program in real school settings.

## Methods

### Participants and procedures

Upon our request, four local boards of education invited all elementary schools in their district to participate in the program; eight principals indicated their interest and consented to participate after receiving a detailed explanation of the study by the research team. Overall, eight public schools participated in the Up2-D2. The current study used feedback sheets filled out by students regarding their perceptions of the program as well as audio data of each lesson recorded by the researchers. Teachers had students complete and return the feedback sheets at the end of each lesson. At the end of this study, we obtained the sheets from 213 children aged 9 to 12 years (4th grade: 39 boys and 47 girls; 5th grade: 46 boys and 42 girls; 6th grade: 26 boys and 13 girls) in seven classes from two schools which comprised of 29.79% of the initial participants. The procedures were conducted in accordance with the ethical standards and approved by the third author’s (A2016-035) institutional research committees and only data that were obtained through an opt-out consent process from the students’ parents were analyzed.

As detailed information on socio-economic status is not usually available from Japanese schools, exact information could not be collected in this regard. Both schools are located in similar middle-class areas of Saitama prefecture, in the suburbs of Tokyo, with homogenous demographics. Before the trials, all classroom teachers attended a local one-day workshop organized by the first author. The teaching plans and visual materials were distributed to them, and they could review the DVD material on which the training session provided by the first author was recorded. Both schools provided the Up2-D2 once per week from September to October.

### Measurements

#### Acceptability

To test the acceptability of the Up2-D2, we developed a feedback sheet containing five questions (except the last lesson, which had four questions; see Table 3). Students completed feedback sheets after each lesson (in most cases, during daily circle time). The first question inquired about the degree of enjoyment of each lesson. The second question was related to the degree that students could understand a “gadget” as a metaphor of cognitive-behavioral skills taught. The third question refers to the degree that they could attain the goal of each lesson. The fourth question was related to experiential understanding while the third question was about conceptual understanding. As shown in Table 3, verbatim expressions of the questions varied according to each lesson. The fifth question asked the degree that they thought they could apply the learned skills to their daily situation. Since the last class was a review of the past lessons, we provided four questions for enjoyable; understandings of all the metaphors; comprehension concerning how to combine learned skills; and promoting daily self-efficacy through all of the lessons. High scores indicated high acceptability and scores of 3 or more can be interpreted as the indices exceeding a threshold of acceptability.

**Table 3:** Acceptability questions for the Up2-D2

No.	Theme	Item example	Scale	Lessons
1	Enjoyment	“Did you enjoy today’s lesson?”	“Enjoyable” = 4, “a little enjoyable” = 3, “a little unenjoyable” = 2, and “unenjoyable” = 1	All lessons
2	Comprehension of a “gadget”	“Did you understand the *XXX*?”	“Understand” = 4, “a little understand” = 3, “not really understand” = 2, and “not understand at all” = 1	All lessons
3	Attainment of the lesson	“Did you differentiate positive and negative emotions?” (Lesson 1)	“Understand” = 4, “a little understand” = 3, “not really understand” = 2, and “not understand at all” = 1	Except the last lesson
		“Did you understand pleasant activities?” (Lesson 2)		
		“Did you understand what words were kind words?” (Lesson 3)		
		“Did you catch the point assertive asking? (Lesson 4)”		
		“Did you understand the relationship between emotion and body? (Lesson 5)”		
		“Did you understand what kinds of strengths existed? (Lesson 6)”		
		“Did you understand the relationship between emotion and thought? (Lesson 7)”		
		“Did you understand what kinds of unhelpful thoughts existed? (Lesson 8)”		
		“Did you understand how to list your difficult situations? (Lesson 9)”		
		“Did you understand how to challenge your difficult situations? (Lesson 10)”		
		“Did you understand the way for problem-solving? (Lesson 11)”		
4	Applicability of the lesson	“Did you understand magnitude of emotions?” (Lesson 1)	“Understand” = 4, “a little understand” = 3, “not really understand” = 2, and “not understand at all” = 1	All lessons
		“Did you find your pleasant activities?” (Lesson 2)		
		“Did you understand four different kind words? (Lesson 3)”		
		“Did you catch the point of assertive declining? (Lesson 4)”		
		“Did you find your favorite relaxation skill?” (Lesson 5)		
		“Did you find your strengths?” (Lesson 6)		
		“Did you understand people think differently even in the same situation?” (Lesson 7)		
		“Did you understand how to cope with the unhelpful thoughts (Lesson 8)”		
		“Did you understand how negative emotion will change if you challenge your difficult situations? (Lesson 9)”		
		“Did you understand how to challenge as small steps (Lesson 10)”		
		“Did you try three steps of problem solving (Lesson 11)”		
		“Did you understand how to combine these gadgets? (Lesson 12)”		
5	Self-efficacy	“Do you think that you can use *XXX* in your daily life to YYY?”	“I think I can do it” = 1, “I think I can do it a little” = 2, I do not think, I can really do it” = 3, and “I never think I can do it” = 4	All lessons

*XXX* a specific gadget name for the lesson, *YYY* a specific application for the lesson, *Up2*-*D2* the Universal Unified Prevention Program for Diverse Disorders

#### Fidelity

We examined the fidelity in real school settings by a test of fidelity when classroom teachers implemented the Up2-D2. All lessons were recorded by IC recorders on site and the archive audio files were kept in the storage of each school. Twenty-one lessons (27.3%) were extracted for evaluation based on a table of random numbers considering the counterbalance of both schools. Research assistants had received rating training though hypothetical lessons independent from the implementation of this study until they obtained over 90% accuracy scores in accordance with the criteria that was set by the first author. Then, they visited each school and independently listened to assigned audio files to evaluate the fidelity of the classes. The first author made evaluation sheets for each class based on the teaching plan. Each sheet had approximately 30 items to evaluate (i.e., range max 24–36 points depending on each lesson), and research assistants confirmed whether a teacher followed the prepared teaching plan.

The evaluation sheets also included what must not be done by teachers in addition to what needs to be done. For example in the group activity in Lesson 2, to fulfill the fidelity criteria, the classroom teachers needed to (1) ask students to generate as many pleasant activities as possible in small groups, (2) have each group express how many activities the students found, and (3) celebrate the group which reported the greatest number of activities; however, teachers should not 4) decide which answers were correct or wrong for each activity, or (5) criticize the group which reported the fewest activities.

## Results

### Acceptability

**Fig. 3:**
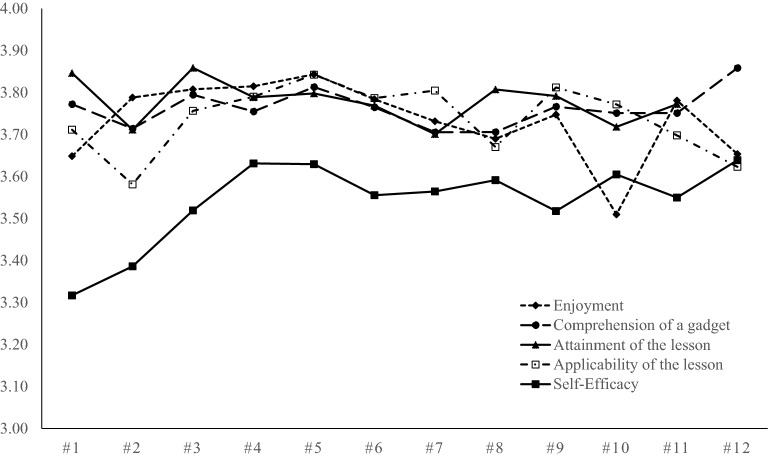
Acceptability of each session of the Up2-D2

Total, 2322 feedback sheets were available (response rate = 90.85%; Table 4). Figure 3 depicts trends of enjoyment, comprehension, attainment, applicability, and self-efficacy from Lessons 1–12 (see also Additional file 1: Table S1). A Tau-U analysis revealed that the trend of self-efficacy was marginally significant, *z* = 1.71, *p* = 0.086. Specifically, self-efficacy had a tendency of gradual increasing through the 12 sessions from 3.32 to 3.64 whereas enjoyment, comprehension, attainment, and applicability were stable and higher than 3.5 for all sessions. Moreover, more than 90% of students who participated in the Up2-D2 responded, “I think I can do it (or a little)” in all lessons (range = 90.59–96.79%) to items of self-efficacy, and 96.10% of them had the confidence to apply leaned cognitive-behavioral skills outside of the classroom immediately after Lesson 12. Whereas Y elementary school showed higher scores for enjoyment than X elementary school in Lessons 10, 11, and 12, after applying the Bonferroni correction (*p* ≤ 0.004), there were no significant differences for comprehension, attainment, applicability, and self-efficacy between the two schools. As a result, all indices of acceptability were above a threshold in all sessions.

**Table 4:** Cross-tabulation of feedback sheets for all lessons

No.	X elementary school			Y elementary school		Total
	4th grade	5th grade	6th grade	4th grade	5th grade	
Lesson 1	34	30	37	47	54	202
Lesson 2	37	31	35	47	53	203
Lesson 3	38	32	34	24	28	156
Lesson 4	37	29	35	45	54	200
Lesson 5	37	28	35	44	54	198
Lesson 6	34	32	36	43	55	200
Lesson 7	35	–	37	43	49	164
Lesson 8	38	32	36	35	47	188
Lesson 9	37	32	34	43	52	198
Lesson 10	38	30	35	47	56	206
Lesson 11	38	29	36	46	53	202
Lesson 12	38	30	36	46	55	205
Total	441	335	426	510	610	2322

We failed to collect feedback sheets of Lesson 7 in 5th grade in X elementary school. The numbers of feedback sheets were combined from two classes in Y elementary school

### Fidelity

Total fidelity of the two schools was 76.2%. It meant that over 75% of the contents of the lessons that the developers had prepared in advance were implemented. X elementary school showed 70.2% and Y elementary school, 82.8%. The current results showed that the one-day workshop provided acceptable fidelity when teachers independently implemented the Up2-D2 in their classrooms given that the fidelity measurements required approximately 30 points to cover during each of the 45-min lessons.

## Discussion

This article described the rationale, components, and preliminary implementation of our new intervention, the Up2-D2. Outwardly, the effort of this study might be considered as usual or ordinary procedures to introduce a new intervention; however, implementation works have rarely appeared in academic papers per se, and it is difficult to share and disseminate such practical wisdom among the field [52]. Since the elaboration tends to be accumulated exclusively within one-party, this study described the effort of implementation explicitly. Therefore, based on the relationships between principles of user-centered design and program features (Fig. 1), we discussed not only current research issues and future perspective of the Up2-D2 but also several challenges in practice and research about universal prevention programs for mental health in schools from the viewpoint of social implementation.

### As a transdiagnostic approach

CBT-based universal prevention programs that typically focus on transdiagnostic approaches have not been well researched. The current study is the first report on the development of a universal transdiagnostic prevention program for both internalizing and externalizing problems in schools. Student feedback indicated that they found the Up2-D2 to be highly enjoyable, understandable, and applicable. In addition, the fidelity of the Up2-D2 was sufficient (approximately 80%), given that the length of teacher training (one-day workshop) was relatively short. Therefore, our findings suggest that the Up2-D2 could be feasible in real school settings when classroom teachers implement the program. Although our findings are encouraging, further trials are needed with larger sample sizes, comprehensive assessments, and rigorous research design for the intervention to be acknowledged as a transdiagnostic program. In particular, future studies should examine the program’s efficacy through a multi-method, multi-informant assessment on multiple domains of psychopathology, such as anxiety, depression, and anger. The current study is a preliminary study and it should be noted that we are aiming to report the efficacy of the Up2-D2 within all eight elementary schools from three prefectures and across five cities. Further trials are necessary to test the efficacy and effectiveness of the Up2-D2.

### Application of teaching plans

As discussed before, teaching plans, instead of ordinary treatment manuals for psychologists, are useful to help teachers ease comprehension and monitoring because of their user-friendliness. Further, teachers are free from special efforts to learn the components of CBT and are prone to adhere to the present program. Indeed, our results showed that all the components were highly understandable for children aged 9–12 years; although, ceiling effects might be seen due to the scale range (i.e., 4-points). Moreover, the fidelity of the program was acceptable given its time-efficiency and methods of evaluation, although it was somewhat lower than those determined in previous trials regarding the prevention of depression in Japan (85–100%) [16]. For example, one of the targeted prevention programs that focused on anxiety and depression in schools required 3 days of training for implementation and used an 11-item questionnaire to measure the competency of cognitive-behavioral practice for evaluation for fidelity [37]. While intensive training is ideal to enhance adherence and fidelity, it is not always feasible in Japanese schools. A nationwide survey revealed that over 99% teachers work over 40 h per a week and an average of 11 h per day [53]. Considering these conditions, it is indispensable to balance cost and benefit between launching the new program and preventive benefit for students. Therefore, one of the ways to *exploit natural constraints* is to rewrite evidence-based psychotherapies into a specific format that provides familiarity, approachability, and learnability, while considering the context. To enhance further program fidelity, consecutive training such as ongoing coaching, continuous consultation, or follow-up training, rather than a one-time training, may be required. Future studies should explore the elements of optimal training in terms of dose and content.

### The role of positive orientation

Concerning positive orientation, the Up2-D2 was developed using educational language and integrated strength works into the lessons. Although face validity is not regarded as formal validity and almost meaningless for researchers [54], it might be helpful for dissemination to the public. Some teachers might feel a cognitive-behavioral program too complicated to learn and misunderstand the program as designed only for children with emotional and behavioral disorders if it excessively emphasized treatment techniques and psychopathology. If the inclusion of strength work convinces school personal to buy into the program, it might be a notable option regarding establishment in educational settings. Moreover, exposure is frequently misunderstood and misused among components of CBT techniques [55, 56], and Japanese children might be reluctant to engage in cognitive restructuring as mentioned previously. Even though, this study showed highly stable satisfaction during the latter part and minimal decrease in enjoyment in building anxiety hierarchy. Therefore, the alignment of the component in this study might be functional for teachers and students to get rid of their hesitation and misunderstandings. However, we need to further investigate the actual reputation of the program and whether potential errors are effectively precluded in the implementation.

### Usability of cartoon story

We anticipated that using cartoons story could contribute to *learnability*, *memorability*, and *low cognitive load* for students. In accord with our hypothesis, the current results suggested that the “gadget” in the Up2-D2 enhanced children’s fun and comprehension. Enjoyment was extremely high for all lessons, except for Lesson 10, as mentioned above. Since Lesson 10 was composed of building an anxiety hierarchy for in vivo exposure to children’s difficult and challenging situations, it is appropriate to consider whether natural deterioration might be suppressed due to the characteristics of the Up2-D2. Moreover, comprehension of the gadgets was stable and high for all components. In general, older adolescents were likely to receive more therapeutic benefits from cognitive-behavioral techniques thanks to more matured cognitive development than younger children [57, 58]. Of interest, components that seem to be relevant to cognitive development such as cognitive restructuring and problem-solving were also highly understandable even when elementary school children aged younger than 13 years participated. Given that teachers generally have no specific knowledge and skills for specific psychotherapies, it is possible that the carton and gadget features of the Up2-D2 can also contribute to *learnability*, *memorability*, and *low cognitive load* for teachers in delivering cognitive-behavioral interventions through a realistic dose of training. Furthermore, the use of cartoons to learn cognitive-behavioral skills can exemplify one of the cultural adaptations to *exploit* ingenious cultural strengths in line with other health education in schools (e.g., stroke education) [59].

### Emphasizing interpersonal practice

Interpersonal practice was another characteristic of the Up2-D2 as well as cultural adaptation along with the cartoon story. All lessons consisted of group activities to *exploit natural constraints* in Japanese elementary educational settings. In addition, social skills trainings were included in Lessons 3 and 4 as an active component. Regarding *satisfaction/reputation*, small-group activities could play a vital role in the program being highly acceptable and well-regarded in school. First, as aforementioned, group social-skills interventions are prevailing CBT approaches among Japanese schools [50]. In a conservative society, it might be more functional to embrace the existing movements since they bear their own benefits; then, one can explore a further integrative approach with extant activities rather than drastic or expulsive ways. Second, teachers in elementary school are used to managing group activities in their classroom. As aforementioned, teachers already use group format and encourage further application in several subjects. Although there have been no empirical studies comparing mono and dual approaches, interpersonal works to enhance the protective environment in each class might lead to utilization of the strength of Japanese culture.

### Limitations and future directions

There are some limitations to be noted, raising future issues related to the social implementation of universal programs for the prevention of diverse mental health problems in schools. First, for future studies using randomized controlled trial design, it is important that future issues, in which domain should be included as an outcome, examine universal transdiagnostic prevention trials. As mentioned above, a multi-method, multi-informant assessment on multiple domains of psychopathology is required to capture intervention gains for a transdiagnostic approach. However, Ollendick and his colleagues criticized that previous studies of transdiagnostic approaches have often focused on psychopathological measurements and never measured how or if changes in these presumed processes mediate treatment outcome [60]. Moreover, universal prevention might promote active personal agency in resilience processes like self-control, self-regulation, or self-efficacy beyond the absence of psychopathological disorders [61]. Besides, previous studies suggested that strength-based interventions in school could promote positive mental health [48, 62]. Therefore, an essential future issue to be explored is how to evaluate gains of universal transdiagnostic preventive interventions focusing on mediators and positive mental health promotion in addition to psychopathological measures.

Second, whereas the study supported social implementation of the Up2-D2, the repeated measurements regarding enjoyment comprehension, attainment, applicability and self-efficacy were used to evaluate each session. More specific and individual assessments could determine more nuanced relationships between the principals and characteristics of the Up2-D2. Since the five characteristics are composed of general features (i.e. teaching plan and cartoon story) as well as more specific aspects to each component (e.g., positive orientation and interpersonal practice), future studies should examine different aspects of social implementation for each lesson considering contextual variables.

Third, this study did not examine effect of the demographics of potential moderators such as gender, age, or school on fidelity or acceptability of the program. Given that the results suggested that a school that had a higher fidelity of the program could produce more enjoyable lessons especially during the latter part of the program, future studies should explore the more direct relationship between fidelity and acceptability. Moreover, this study only used students’ reports for the evaluation of acceptability. Teachers’ subjective reports on usability and feasibility should be examined in future studies. In addition, acceptability from parents and stake holders could be useful information for social implementation. Therefore, an important future task would be the identification of factors that contribute to acceptability, and to conduct multi-level analyses including these variables.

## Supplementary information

**Additional file 1: Table S1.** Means and standard deviations of acceptability of the Up2-D2 in children (*N* = 213).

## Data Availability

The datasets used and/or analyzed during the current study are available from the corresponding author on reasonable request.

## Abbreviations

ADHD: attention-deficit/hyperactivity disorders
CBT: cognitive-behavioral therapy
ES: effect size
UP: Unified Protocol for the Transdiagnostic Treatment of Emotional Disorders
Up2-D2: Universal Unified Prevention Program for Diverse Disorders
