# Supplementary material for: Developing the universal unified prevention program for diverse disorders for school-aged children
Source: Child Adolesc Psychiatry Ment Health. 2019 Nov 13;13:44. doi: 10.1186/s13034-019-0303-2 (PMC6852986; doi:10.1186/s13034-019-0303-2)
Supplement: Supplementary file 1 — Additional file 1: Table S1. Means and standard deviations of acceptability of the Up2-D2 in children (N = 213). [file 13034_2019_303_MOESM1_ESM.docx]

**Table S1. Means and standard deviations of acceptability of the Up2-D2 in children (*N* = 213).**

|  | # 1 | # 2 | # 3 | # 4 | # 5 | # 6 | # 7 | # 8 | # 9 | # 10 | # 11 | # 12 |
| --- | --- | --- | --- | --- | --- | --- | --- | --- | --- | --- | --- | --- |
| Enjoyment | 3.65 | 3.79 | 3.81 | 3.82 | 3.84 | 3.78 | 3.73 | 3.69 | 3.75 | 3.51 | 3.78 | 3.65 |
|  | (0.59) | (0.45) | (0.41) | (0.41) | (0.39) | (0.52) | (0.56) | (0.59) | (0.50) | (0.88) | (0.48) | (0.70) |
| Gadget comprehension | 3.77 | 3.71 | 3.79 | 3.76 | 3.81 | 3.77 | 3.71 | 3.71 | 3.77 | 3.75 | 3.75 | 3.86 |
|  | (0.44) | (0.51) | (0.46) | (0.49) | (0.45) | (0.47) | (0.56) | (0.52) | (0.47) | (0.51) | (0.50) | (0.40) |
| Lesson attainment | 3.85 | 3.71 | 3.86 | 3.79 | 3.80 | 3.77 | 3.70 | 3.81 | 3.79 | 3.72 | 3.77 | - |
|  | (0.40) | (0.50) | (0.40) | (0.45) | (0.46) | (0.50) | (0.55) | (0.45) | (0.52) | (0.57) | (0.49) |  |
| Lesson applicability | 3.71 | 3.58 | 3.76 | 3.79 | 3.84 | 3.79 | 3.80 | 3.67 | 3.81 | 3.77 | 3.70 | 3.62 |
|  | (0.48) | (0.61) | (0.50) | (0.44) | (0.40) | (0.53) | (0.44) | (0.58) | (0.40) | (0.49) | (0.57) | (0.71) |
| Self-efficacy | 3.32 | 3.39 | 3.52 | 3.63 | 3.63 | 3.56 | 3.56 | 3.59 | 3.52 | 3.60 | 3.55 | 3.64 |
|  | (0.65) | (0.68) | (0.60) | (0.63) | (0.68) | (0.72) | (0.68) | (0.62) | (0.70) | (0.67) | (0.75) | (0.57) |

*Note.* Up2-D2 = the Universal Unified Prevention Program for Diverse Disorders; 4 = highly acceptable, 1 = non-acceptable.
